# Alzheimer's disease-causing presenilin-1 mutations have deleterious effects on mitochondrial function

**DOI:** 10.7150/thno.59776

**Published:** 2021-08-17

**Authors:** Jihoon Han, Heejin Park, Chinmoyee Maharana, A-Ryeong Gwon, Jinsu Park, Seung Hyun Baek, Han-Gyu Bae, Yoonsuk Cho, Hark Kyun Kim, Jae Hoon Sul, Jeongmi Lee, Eunae Kim, Junsik Kim, Yongeun Cho, Sunyoung Park, Leon F. Palomera, Thiruma V. Arumugam, Mark P. Mattson, Dong-Gyu Jo

**Affiliations:** 1 School of Pharmacy, Sungkyunkwan University, Suwon 16419, Korea.; 2 Laboratory of Neurosciences, National Institute on Aging, National Institutes of Health, Baltimore, MD 21224, USA.; 3 Department of Zoology, University of Jammu, Jammu Tawi - 180006, India.; 4 School of Life Sciences, La Trobe University, Bundoora, Victoria, Australia.; 5 Department of Neuroscience, Johns Hopkins University, Baltimore, MD 21205, USA.; 6 Samsung Advanced Institute for Health Sciences and Technology, Sungkyunkwan University, Seoul 06351, Korea.; 7 Biomedical Institute for Convergence, Sungkyunkwan University, Suwon 16419, Korea.

**Keywords:** Alzheimer's disease, Presenilin-1, Mitochondria, MAMs, ATL2

## Abstract

Mitochondrial dysfunction and oxidative stress are frequently observed in the early stages of Alzheimer's disease (AD). Studies have shown that presenilin-1 (PS1), the catalytic subunit of γ-secretase whose mutation is linked to familial AD (FAD), localizes to the mitochondrial membrane and regulates its homeostasis. Thus, we investigated how five *PS1* mutations (A431E, E280A, H163R, M146V, and Δexon9) observed in FAD affect mitochondrial functions.

**Methods:** We used H4 glioblastoma cell lines genetically engineered to inducibly express either the wild-type PS1 or one of the five PS1 mutants in order to examine mitochondrial morphology, dynamics, membrane potential, ATP production, mitochondria-associated endoplasmic reticulum (ER) membranes (MAMs), oxidative stress, and bioenergetics. Furthermore, we used brains of PS1M146V knock-in mice, 3xTg-AD mice, and human AD patients in order to investigate the role of PS1 in regulating MAMs formation.

**Results:** Each PS1 mutant exhibited slightly different mitochondrial dysfunction. Δexon9 mutant induced mitochondrial fragmentation while A431E, E280A, H163R, and M146V mutants increased MAMs formation. A431E, E280A, M146V, and Δexon9 mutants also induced mitochondrial ROS production. A431E mutant impaired both complex I and peroxidase activity while M146V mutant only impaired peroxidase activity. All PS1 mutants compromised mitochondrial membrane potential and cellular ATP levels were reduced by A431E, M146V, and Δexon9 mutants. Through comparative profiling of hippocampal gene expression in PS1M146V knock-in mice, we found that PS1M146V upregulates Atlastin 2 (ATL2) expression level, which increases ER-mitochondria contacts. Down-regulation of ATL2 after PS1 mutant induction rescued abnormally elevated ER-mitochondria interactions back to the normal level. Moreover, ATL2 expression levels were significantly elevated in the brains of 3xTg-AD mice and AD patients.

**Conclusions:** Overall, our findings suggest that each of the five FAD-linked *PS1* mutations has a deleterious effect on mitochondrial functions in a variety of ways. The adverse effects of PS1 mutations on mitochondria may contribute to MAMs formation and oxidative stress resulting in an accelerated age of disease onset in people harboring mutant PS1.

## Introduction

Alzheimer's disease (AD) is the most common form of dementia for which there is no cure or effective treatment. AD is characterized by pathological phenotypes that include β-amyloid (Aβ) peptide accumulation in the brain, intracellular tau aggregation, chronic neuroinflammation, and mitochondrial dysfunction 1,2. For the past two decades, Aβ accumulation and tau aggregation have been the central focus and targets for AD research and drug development. Unfortunately, most drugs targeting these two factors have failed. Therefore, extensive studies are underway to overcome AD with a multifaceted approach that targets the pathological phenotypes of AD other than Aβ accumulation and tau aggregation 3.

The most critical risk factor for developing AD is aging, which is a process that results in a gradual and progressive decline in physiological functions, eventually leading to many pathological conditions such as cancer, arthritis, stroke, and neurodegenerative diseases 4-9. Oxygen radicals (reactive oxygen species; ROS) produced from normal metabolism could cause irreversible cell damage and aging-related degenerative diseases 10,11. Mitochondria are considered the primary source and a target of free radicals and mitochondrial damage accumulation can impair respiratory chain function and increase ROS production 12. Moreover, it has been reported that the progressive mitochondria membrane damage caused by free radicals could result in an increase in peroxide generation and an age-related decrease in functionally competent mitochondria and cellular ATP production 13. The role of mitochondria in aging was further emphasized as more evidence suggested that somatic mutation accumulation in mitochondrial DNA (mtDNA) is a significant contributor to human aging and degenerative disease 14.

Since aging is the most significant risk factor in developing AD, it is logical to consider that mitochondrial dysfunction contributes to the disease process. In fact, studies have shown that mitochondrial dysfunction and oxidative stress play a significant role in early AD pathogenesis 15-17. Moreover, it has been reported that oxidative damage occurs even before Aβ plaque formation, which strongly supports a causative role of mitochondrial dysfunction and oxidative stress in AD 18-20. Since the amyloid cascade hypothesis, which states that the primary event in AD neurodegeneration is the production of Aβ, is most viable in the case of familial AD (FAD) but not in sporadic AD (SAD), a mitochondrial cascade hypothesis was proposed in 2004 21-24. This theory suggests that mitochondrial dysfunction represents primary pathology in SAD, driving both Aβ plaque and neurofibrillary tangle formation 24. It has gained support as numerous mitochondrial dysfunctions were reported in the context of AD such as abnormal mitochondrial morphology, disrupted oxidative phosphorylation, increased ROS production and mitochondria-associated endoplasmic recticulum (ER)-mitochondria membranes (MAMs), impaired mitochondrial biogenesis, etc. 25-29.

Presenilin-1 (PS1), an aspartyl protease, is the catalytic subunit of γ-secretase that mediates cleavage of type I transmembrane proteins, including amyloid precursor protein (APP) within its transmembrane region 30. Sequential cleavage of APP by β-secretase and γ-secretase produces Aβ, which is deposited as plaques in the brains of AD patients 31,32. More than 300 mutations within the entire sequence of PS1 have been identified thus far, and most of them are associated with early-onset FAD. These FAD-linked PS1 mutations induce a consistent PS1 conformation change, which leads to a shift in the Aβ42/40 ratio 33,34. Moreover, during normal aging and in SAD, the conformation of endogenous PS1 changes to a “closed” conformation, which is similar to that observed in FAD-linked PS1 mutants 20, 35. Therefore, PS1 plays a critical role in the pathogenesis of both FAD and SAD. PS1 is known to be localized to numerous compartments of the cell including ER, Golgi, nuclear envelope, endosomes, lysosomes, the plasma membrane, and mitochondria 36,37. More specifically, in 2009, it was reported that PS1 is highly enriched in MAMs, subdomains of the ER in contact with mitochondria 37. It is known to play a critical role in phospholipid biosynthesis, cholesterol esterification, calcium transport, and homeostasis of mitochondria and ER 37,38.

**Figure 1:**
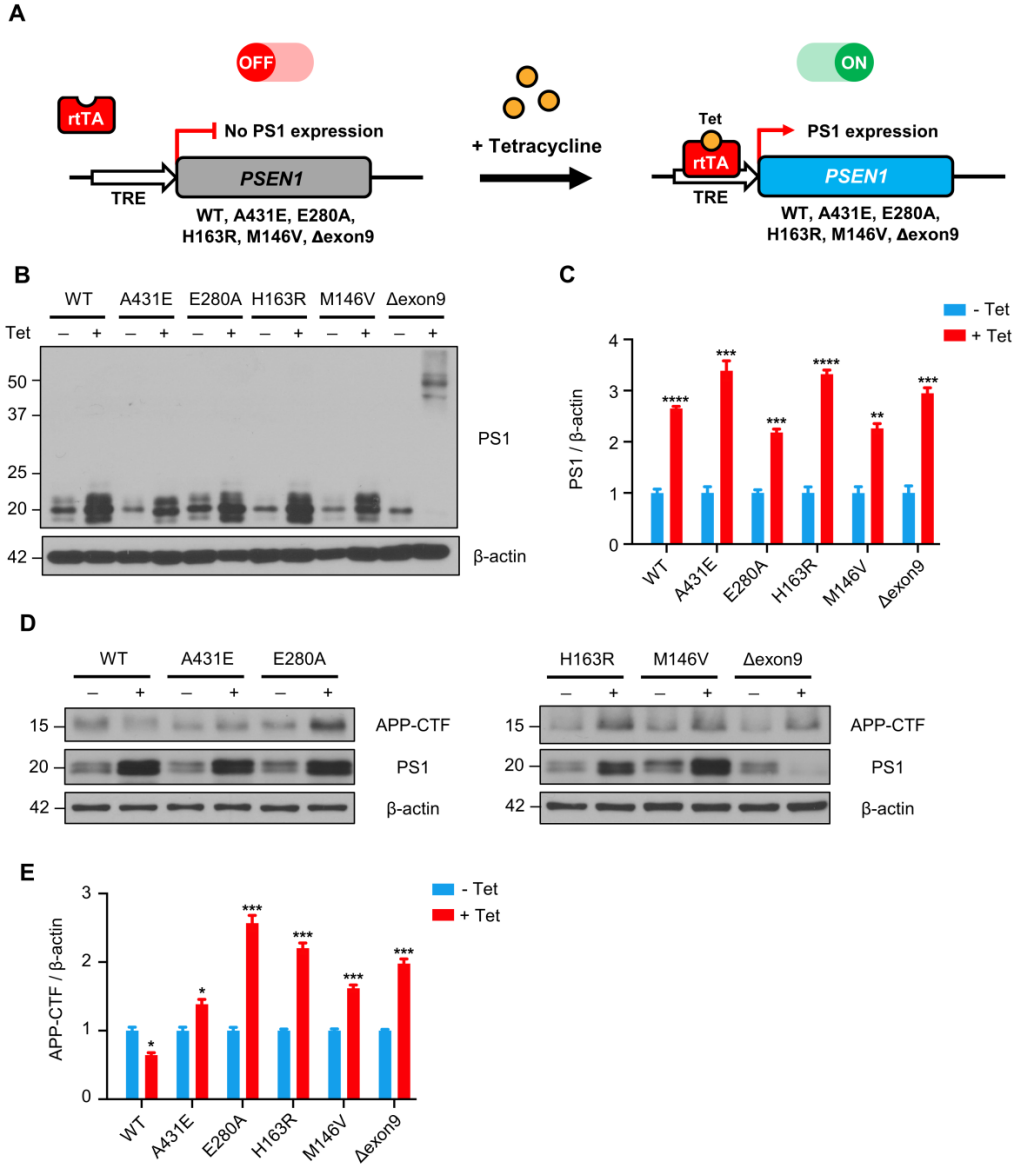
** APP-CTFs are accumulated in H4^PS1^ cell lines upon PS1 mutant induction. (A)** A schematic diagram of Tet-on inducible system for H4^PS1^ cell line. **(B)** A representative western blot of PS1 in H4^PS1^ cell lines before and after tetracycline (100 ng/ml) treatment. **(C)** Quantification of PS1 mutant expression in (B). n = 3; **P < 0.01, ***P < 0.001, ****P < 0.0001; Student's *t*-test (two-tailed). **(D)** A representative western blot of APP-CTFs in H4^PS1^ cell lines before and after tetracycline (100 ng/ml) treatment. **(E)** Quantification of APP-CTFs in (D). n = 3; *P < 0.05, **P < 0.01, ***P < 0.001; Student's *t*-test (two-tailed). The values shown indicate the means ± SEM.

As PS1 is highly enriched in MAM, we investigated the effect of five different *PS1* mutations identified in FAD (A431E, E280A, H163R, M146V, Δexon9) on mitochondrial functions. We used six H4 glioblastoma cell lines, each genetically engineered to express either PS1 wild-type (WT) or one of the five PS1 mutants in an inducible manner (Figure 1A). By utilizing confocal microscopy, transmission electron microscopy (TEM), and Seahorse XF24 extracellular flux analyzer, we observed the effect of each PS1 mutation on mitochondrial morphology, ER-mitochondria interaction, and mitochondrial bioenergetics. Furthermore, we assessed the impact of each *PS1* mutation on mitochondrial ROS production, membrane potential, ATP production, complex I activity, and peroxidase activity. Each mutant cell line exhibited a different degree of mitochondrial dysfunctions, but in general, all mutations except for Δexon9 showed an increase in ER-mitochondria interaction. Gene expression profiling in the hippocampus samples from wild-type and PS1M146V knock-in mice revealed a critical role of Atlastin 2 (ATL2) in the formation of ER-mitochondria contact sites. Moreover, significantly elevated ATL2 expression levels were observed in the brains of both 3xTg-AD mice and AD patients. Overall, our findings suggest a deleterious effect of *PS1* mutations identified in FAD on mitochondrial functions.

## Methods

### Animals

Seven-month-old homozygous PS1M146V knock-in mice and their wild-type littermates of the same sex (male) and genetic background (C57BL/6) were maintained under pathogen-free conditions on a 12 h light-dark cycle with continuous access to food and water. All procedures were reviewed and approved by the NIA Animal Care and Use Committee. Seven and twelve-month-old 3xTg-AD mice (APP Swedish, MAPT P301L, PSEN1 M146V) and their wild-type littermates of the same genetic background (C57BL/6) were maintained under pathogen-free conditions on a 12 h light-dark cycle with continuous access to food and water. All experiments and procedures were approved by the Institutional Animal Care and Use Committee of Sungkyunkwan University.

### Human brain specimens

Inferior parietal lobule specimens from the brains of three AD patients and three age-matched control subjects that had been enrolled in the University of Kentucky Alzheimer's Disease Center Autopsy Program were used for this study. At autopsy, tissue specimens were rapidly removed, frozen, and stored at -80 °C.

### Allen Brain Institute Aging, Dementia, and TBI Database

This database contains RNAseq-derived transcriptome data from 107 subjects obtained from the Adult Change in Thought (ACT) cohort. A detailed description of tissue collection, tissue processing, and quantitative data generation is available in the dataset documentation (http://help.brain-map.org/display/aging/Documentation). RNAseq data from frontal white matter (FWM) of each subject were used for our gene expression level analysis.

### Cell culture

*Homo sapiens* brain neuroglioma H4 cell lines stably expressing wild type (WT) PS1 or EOFAD linked PS1 mutants (A431E, E280A, H163R, M146V or Δexon9) under the control of the tetracycline repressor element were a generous gift from Brandon Wustman and Anthony Stevens from Amicus Therapeutics, Inc (1 Cedar Brrok Drive, Cranbury, NJ USA). Cells were cultured in Dulbecco's modified Eagle medium (DMEM) (Corning, #10-013-CV) supplemented with 10% fetal bovine serum (Gibco, #12483020), 50 µg/ml Zeocin (Invitrogen, #R25001), and 2.5 µg/ml Blasticidin (Sigma-Aldrich, #15205). Cultures were maintained at 37 °C with 95% O_2_ and 5% CO_2_ 39.

### Tissue sample preparation

Tissue sample preparation was performed as described previously 40. Briefly, mice were anesthetized with Zoletil (Virbac) and Rompun (Bayer), and perfused with phosphate-buffered saline (PBS) (PBS; P3813) with 0.9% NaCl concentration. Brains were dissected to separate hippocampi, which were flash-frozen in liquid nitrogen and stored at -80 °C until analysis.

### Western blot analysis

Cells were collected using T-PER^TM^ tissue protein extraction reagent (Thermo Scientific, #78510) with protease/phosphatase inhibitor cocktail (Biovision, #K276-1) and western blot analysis was performed as described previously 41. Briefly, proteins were quantified with a BCA protein assay kit (Thermo Fisher Scientific, #23225) using a xMark microplate spectrophotometer (Bio-Rad, Hercules, CA, USA) in the BIORP of Korea Basic Science Institute (KBSI). Then, samples were resolved on a SDS-polyacrylamide gel and transferred to a PVDF membrane. Depending on the molecular weight of target proteins, different percentage of SDS-polyacrylamide gel was used. For ATL1, ATL2, ATL3, Tau-13, BACE1, MFN2, OPA1, DRP1, and β-actin, 8% SDS-polyacrylamide gel was used. For PS1 and APP-CTF, 12% SDS-polyacrylamide gel was used. Blots were blocked in 5% non-fat dry milk for 1 h at room temperature before incubating overnight with the primary antibodies against ATL1 (PA5-85682, Invitrogen), ATL2 (PA5-90788, Invitrogen), ATL3 (PA5-88408, Invitrogen), PS1 (5643, Cell Signaling), APP-CTF (A8717, Sigma), Tau-13 (835201, Biolegend), BACE1 (5606, Cell Signaling), MFN2 (sc-100560, Santa Cruz), OPA1 (612606, BD Biosciences), DRP1 (sc-32898, Santa Cruz), and β-actin (A5316, Sigma-Aldrich). ATL1, ATL2, ATL3, PS1, APP-CTF, Tau-13, BACE1, and OPA1 antibodies were used at dilution of 1:1000. MFN2 and DRP1 antibodies were used at dilution of 1:200. β-actin antibody was used at dilution of 1:10000. The membranes were incubated with HRP-conjugated secondary antibody for 1 h at room temperature, and the signal was detected using ECL solution (Pierce, Rockford, IL, USA). Quantification of western blot bands was performed using ImageJ program.

### ER and mitochondria imaging analysis

H4^PS1^ cell lines were grown in DMEM supplemented with 10% fetal bovine serum, 50 µg/ml Zeocin, and 2.5 µg/ml Blasticidin. For PS1 mutant induction, each cell line was treated with 100 ng/ml of tetracycline (Sigma-Aldrich, #T7660) for 5 days 39. Cells were passaged and plated on Nunc Lab-Tek Chambered coverglass (Thermo Fisher Scientific, #155361) 24 h before imaging. Cells were incubated in Hank's Balanced Salt Solution (HBSS) containing 100 nM MitoTracker^TM^ Red (Thermo Fisher Scientific, #M7512) and 1 µM ER-Tracker^TM^ Green (Thermo Fisher Scientific, #E34251) for 20 min at 37 °C in a 5% CO_2_ atmosphere, from stocks prepared according to the manufacturer's instructions. For fixation, cells were washed with PBS twice, for 5 min each time, fixed with 4% paraformaldehyde (PFA) for 10 min, and washed in PBS twice (5 min each time). The slides were mounted in Antifade Mounting Medium with DAPI (VECTASHIELD, #H-1200). For live-cell imaging, HBSS was replaced with a fresh probe-free medium after 20 min incubation. Confocal imaging was performed using a LSM700 (Carl Zeiss, Göttingen, Germany). 405-nm laser was used for DAPI (Ex/Em = 365/468). 488-nm laser was used for ER-Tracker^TM^ Green (Ex/Em = 504/511) and 555-nm laser was used for MitoTracker^TM^ Red (Ex/Em = 579/599). Images were acquired, and the fluorescence intensity profile and weighted colocalization coefficient were analyzed using the Zeiss ZEN software. The weighted colocalization coefficients were calculated using the same equation as the Mander's colocalization coefficients, but the value for each pixel was equal to its intensity value.

### Transmission electron ultrastructural analysis

Each H4^PS1^ cell line was fixed, processed, and visualized as previously described 42. Briefly, samples were prepared using ultrathin sectioning (80 nm) and stained using uranyl acetate and sodium bismuth. The sections were examined using a transmission electron microscope (JEM-1010, JEOL, Tokyo, Japan).

### Mitochondrial superoxide detection

Mitochondrial superoxide was detected using the fluorescent MitoSOX^TM^ Red (Invitrogen, #M36008). Cells were incubated with 2 μM MitoSOX^TM^ Red for 30 min at 37 °C in a 5% CO_2_ atmosphere before washing with PBS. The fluorescence was detected using confocal microscopy (Carl Zeiss LSM700). 405-nm laser was used for Hoechst (Ex/Em = 361/497) and 555-nm laser was used for MitoSOX^TM^ Red (Ex/Em = 510/580).

### Complex I activity

Mitochondrial complex I activity of each H4^PS1^ cell line was determined using the Complex I Enzyme Activity Microplate Assay Kit (Abcam, #ab109721) according to the kit instructions. Briefly, 200 μg of protein was incubated in 'incubation buffer' for 3 h at room temperature and washed three times with '1× buffer'. Assay buffer was added, and samples were analyzed on a Synergy HTX Multi-Mode Microplate Reader (BioTek Instruments, Inc, USA) (Abs: 450 nm, at 45 sec intervals for 30 min, shaking sample between each reading).

### Peroxidase activity

Peroxidase activity of each H4^PS1^ cell line was determined using the EZ-Hydrogen Peroxide/Peroxidase assay kit (DoGenBio, #DG-PER500) according to the manufacturer's instructions. Briefly, 50 µl of sample and HRP standard solution was incubated with 50 ul of Oxi-Probe/H_2_O_2_ working solution for 30 min at room temperature in the dark. Each well was analyzed on a Synergy HTX Multi-Mode Microplate Reader (Abs: 560 nm).

### Seahorse analysis

The Seahorse analyzer XF24 (Agilent, Santa Clara, USA) was used according to the kit instructions to monitor the oxygen consumption rate (OCR) in real-time. Briefly, two days before the experiment, each H4^PS1^ cell line was seeded in a XF24 cell culture plate. One day prior to the experiment, 1 ml of XF calibrator was added to each well of the XF cartridge and incubated overnight at 37 °C in humidified atmosphere and 0% CO_2_. Thirty minutes prior to the experiment, cells were washed with PBS, and 625 μl of respective XF assay medium was added per well before incubating for 30 min at 37°C in a humidified atmosphere and 0% CO_2_. For the XF Cell Mito Stress analysis, XF assay medium was supplemented with 5 mM glucose and 2 mM glutamine. After a 15 min equilibration time, OCR was measured every 8.30 min (after 3 min mixing, 2 min wait, 3.30 min measurement), 4 times after the addition of respective compounds. The different compounds were added to the injection ports of the XF cartridge in 10x of final concentration, and they were diluted in XF assay medium before the experiment.

### Microarray analysis

Mice were sacrificed by cervical dislocation and hippocampus was removed and flashes frozen. The tissue was processed with a Bead Beater (Bio-Spec, Bartlesville, OK, USA) and RNA was purified using RNEasy Mini Kits (Qiagen, Valencia, CA, USA). Purified RNA was evaluated for quality and quantity with a Bioanalyzer (Agilent Technologies, Palo Alto, CA, USA). Probe preparation and hybridization were done as previously described 43. Briefly, 5 μg of each RNA sample was used in a PCR reaction with 32^P^-dCTP (Valeant, Costa Mesa, CA, USA). Radiolabeled cDNA was allowed to hybridize overnight at 43 °C to the mouse NIA 17K cDNA filters 44. The hybridized filters were then washed and placed under imaging screens for three days. The images were developed and scanned. The data were extracted using ArrayPro Software (Media Cybernetics, San Diego, CA, USA).

### Differentially expressed genes (DEGs) selection & functional analysis

All data were processed by z-score transformation 45. Genes whose average intensity between the two conditions was greater than zero were first identified. Then, the genes with z-ratios greater than or equal to 1.5, less than or equal to -1.5, and *P* value < 0.05 were selected as DEGs. The z-ratio is a measure of fold change between comparisons 45. All gene lists were annotated with RefSeq-release23 and Unigene database9. The heat map for the gene expression values and the volcano plots for the z-ratio and *P* values between the selected samples were drawn by in-house R scripts. The GO terms enriched in the DEGs were determined using g:Profiler (ver 0.6.7).

### Transient transfection

Cells were seeded in 6-well dishes at 70% confluence and transfected with siRNA against ATL2 (Bioneer, Daejeon, South Korea) using OptiMEM and INTERFERin (Polyplus-transfection), according to the manufacturer's instructions. Transfected cells were maintained for 24 h prior to further experiment. The sequences of siRNA are as follow:

- ATL2 sense, 5'-UAG AGU UUG UUA CAG ACU G-3';
- ATL2 antisense, 5'-CAG UCU GUA ACA AAC UCU A-3'.

### Quantitative real-time PCR (qPCR)

Total RNA was extracted by RNAiso plus (TaKaRa, Shiga, Japan) from H4^PS1^ cell lines and reverse transcribed into cDNA using PrimeScript RT Master Mix (TaKaRa). qPCR was performed to detect mRNA expression of *ATL1*, *ATL2*, *ATL3* by TB Green PCR Kit (TaKaRa), and GAPDH was used as a reference gene. The qPCR primer sequences used are as follow:

- *ATL1* forward, 5'-CCC TGT GCA CTT GGG CAT AT-3';
- *ATL1* reverse, 5'-TTG TAC AAA GCC TGG TCC CAC-3';
- *ATL2* forward, 5'-TTG CCA CAT CCT GGT CTT AAA-3';
- *ATL2* reverse, 5'-GCA GCA ATG GAA CCA GAT TT-3';
- *ATL3* forward, 5'-ACA AGC CCT GAC TTT GAT GG-3';
- *ATL3* reverse, 5'-TGC AGC TGC TAA GTT GTT GG-3';
- *GAPDH* forward, 5'-AGC CAC ATC GCT CAG ACA C-3';
- *GAPDH* reverse, 5'-GCC CAA TAC GAC CAA ATC C-3'.

### Intracellular ROS measurements

Intracellular ROS levels were measured using a fluorescent dye, chloromethyl 2',7'-dichlorofluorescein diacetate (CM-H_2_DCFDA) (Invitrogen, #C6827), which is converted to the highly fluorescent 2',7'-dichlorofluorescein (DCF) in the presence of an oxidant. Briefly, each H4^PS1^ cell line was plated in 96-well plates. Twenty-four hours later, cells were incubated with CM-H_2_DCFDA (5 μM) in serum-free medium for 30 min. The fluorescence intensity was detected using a Victor X3 plate reader (Perkin Elmer, USA) according to the manufacturer's protocol (Ex/Em = 492/527).

### Mitochondrial membrane potential and ATP level measurement

Mitochondrial membrane potential was measured in H4^PS1^ cell lines in a 96-well plate with either JC-1 (5,5',6,6'-tetrachloro-1,1',3,3'-tetraethylbenzimidazolylcarbocyanine iodide) (BD Biosciences, #551302) or TMRM (tetramethylrhodamine, methyl ester, perchlorate). The fluorescence intensity for aggregates and monomeric forms of JC-1 was detected using a TECAN Infinite 200 multifunctional microplate reader (TECAN, Switzerland) according to the manufacturer's protocol (JC-1 aggregates: Ex/Em = 525/590; JC-1 monomers: Ex/Em = 490/530). The fluorescence intensity for TMRM was detected using Synergy NEO multi-mode microplate reader (BioTek Instruments, USA) according to the manufacturer's protocol (Ex/Em = 548/574). Total cellular ATP levels were detected using an ATP bioluminescence detection kit (Promega, #TB267) according to the manufacturer's protocol.

### Statistical analysis

All statistical analyses were performed using GraphPad Prism 8.0 software (GraphPad, La Jolla, CA, USA). All data are presented as mean ± standard error (SEM). Depending upon the variables, one-way or two-way analysis of variance (ANOVA) with Bonferroni post-hoc test was used to compare group differences. Student *t*-test (two-tailed) was used to compare each H4^PS1^ cell line before and after PS1 induction. A *P*-value < 0.05 was considered statistically significant.

## Results

### APP-CTFs are accumulated in H4^PS1^ cell lines upon PS1 mutant induction

Since PS1 is the catalytic subunit of γ-secretase, which cleaves type 1 transmembrane proteins, we first examined the effect of each PS1 mutant on γ-secretase activity by detecting APP C-terminal fragments (APP-CTFs). Each PS1 mutant was successfully induced by 100 ng/ml of tetracycline treatment and the degree of overexpression was observed to be 2.2 to 3.4-fold (Figure 1B-C). It is worth nothing that in H4^PS1Δexon9^ cell line, 44 kDa full-length PS1 was accumulated upon tetracycline treatment. This is because deletion of exon 9, which contains endoproteolysis site, prevents the cleavage of full-length PS1. Furthermore, the endogenous PS1 was diminished in H4^PS1Δexon9^ cell line upon tetracycline treatment (Figure 1B). This is consistent with the findings from previous study which reported that overexpression of exogenous PS1 replaces endogenous PS1 46. This replacement allowed us to exclude any potential effects of endogenous PS1 in the induced H4^PS1^ cell lines. Upon induction, we observed an accumulation of APP-CTFs in all five H4^PS1^ mutant cell lines, indicating decreased γ-secretase activity (Figure 1D-E). This result is consistent with the findings of earlier studies that reported attenuated γ-secretase activity upon expression of FAD-linked PS1 mutant proteins 47-52.

### Fragmented mitochondria are accumulated in H4^PS1Δexon9^ cell line upon PS1 mutant induction

A mitochondrion is one of the most dynamic cellular organelles; it can change size, shape, and position 53. Mitochondria undergo fusion and fission to maintain their functionality under metabolic or environmental stresses 54. Fusion integrates the contents of different mitochondria to alleviate stress, while fission manages clearance of damaged mitochondria and the production of new mitochondria 54. Increased mitochondrial fission has been documented in neurons in several neurodegenerative disorders, including AD 55.

**Figure 2:**
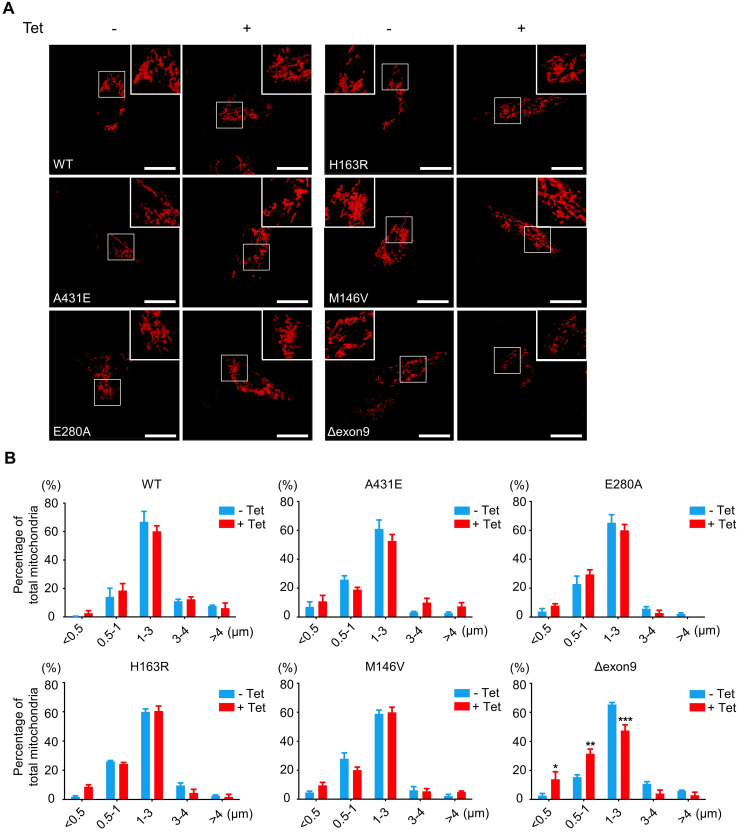
** Fragmented mitochondria are increased in the H4^PS1Δexon9^ cell line upon induction. (A)** Representative confocal images of Mito-Tracker (red) labeled H4^PS1^ cell lines before and after tetracycline (100 ng/ml) treatment. Scale bar, 20 µm. **(B)** Quantification of mitochondrial lengths in H4^PS1^ cell lines before and after tetracycline (100 ng/ml) treatment. For each independent imaging experiment, 8-12 cells were analyzed. Number of mitochondria per cell 48-121, n = 3; *P < 0.05*,* **P < 0.01, ***P < 0.001; Two-way ANOVA with Bonferroni post-hoc test. The values shown indicate the means ± SEM.

Thus, we investigated whether *PS1* mutations affect mitochondrial fusion or fission by examining the size and shape of mitochondria in each H4^PS1^ cell line. Before PS1 mutant induction, all six H4^PS1^ cell lines exhibited filamentous sausage-like mitochondria (Figure 2A). After PS1 mutant induction, the H4^PS1Δexon9^ cell line showed an increase in fragmented mitochondria, which were shortened, punctate, and spherical (Figure 2A). When the size of mitochondria was measured using confocal microscopy, there was a significant increase in fragmented mitochondria in the H4^PS1Δexon9^ cell line after induction (Figure 2B). No significant mitochondrial size difference was observed in other H4^PS1^ cell lines before and after PS1 mutant induction. Moreover, the H4^Δexon9^ cell line exhibited relatively aggregated mitochondria in the cytosol upon induction when mitochondrial distribution was observed using z-stack imaging (Figure S1A). Next, we examined the expression level of proteins related to mitochondrial dynamics like optic atrophy1 (OPA1), mitofusin 2 (MFN2), and GTPase dynamin-related protein 1 (DRP1). OPA1 and MFN2 mediate mitochondrial fusion, while DRP1 mediates mitochondrial fission 54,56. Drp1 activity and mitochondrial fragmentation are reportedly elevated in the brains of SAD 57-59. An earlier study reported that DRP1 inhibition could ameliorate Aβ-mediated mitochondrial dysfunction 60. Surprisingly, the DRP1 and MFN2 expression levels were significantly decreased after PS1 mutant induction in H4^PS1Δexon9^ cell line while there was no difference in OPA1 expression level (Figure S1B-E).

### ER-mitochondria contacts are increased in H4^PS1A431E^, H4^PS1E280A^, H4^PS1H163R^, and H4^PS1M146V^ cell lines upon PS1 mutant induction

**Figure 3:**
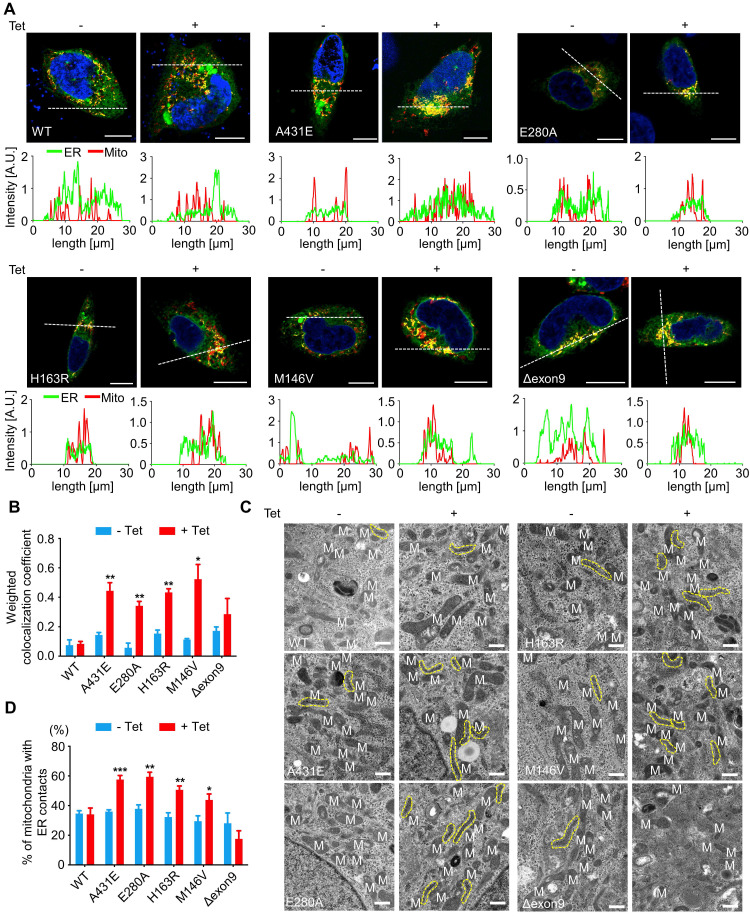
** ER-mitochondria interaction is elevated in H4^PS1A431E^, H4^PS1E280A^, H4^PS1H163R^, and H4^PS1M146V^ cell lines upon induction. (A)** Representative confocal images and line scan analysis of Mito-Tracker (red) and ER-Tracker (green) labeled H4^PS1^ cells before and after tetracycline (100 ng/ml) treatment. Scale bar, 10 µm. White dotted line marks fluorescence intensity profile position. **(B)** The weighted colocalization coefficient between mitochondria and ER in H4^PS1^ cell lines before and after tetracycline (100 ng/ml) treatment. For each independent imaging experiment, 10-14 cells were analyzed. n = 3; *P < 0.05*,* **P < 0.01, ***P < 0.001; Student's *t*-test (two-tailed). **(C)** Representative transmission electron microscope images of H4^PS1^ cell lines before and after tetracycline (100 ng/ml) treatment. The yellow dotted line indicates mitochondria contacting ER. Scale bar, 0.5 µm. **(D)** Quantification of mitochondria in contact with ER in H4^PS1^ cell lines before and after tetracycline (100 ng/ml) treatment. For each independent imaging experiment, 5-10 cells were analyzed. Number of mitochondria per cell 10-35, n = 4; *P < 0.05*,* **P < 0.01, ***P < 0.001; Student's *t*-test (two-tailed). The values shown indicate the means ± SEM.

PS1 and PS2 have been observed in numerous subcellular compartments: ER, Golgi, plasma membrane, nuclear envelop, endosomes, lysosome, and mitochondria 61-66. Specifically, they are highly enriched in MAMs, an ER subcompartment that is physically connected to mitochondria 67. MAMs allow direct communication between the ER and mitochondria. Since this communication plays an essential role in determining cell fate by controlling the functioning of all other intracellular compartments, we used confocal microscopy to examine the effect of each *PS1* mutation on the interaction between ER and mitochondria. After induction, H4^PS1A431E^, H4^PS1E280A^, H4^PS1H163R^, and H4^PS1M146V^ cell lines exhibited increased ER-mitochondria colocalization. This change was not detected in H4^PS1WT^ and H4^PS1Δexon9^ cell lines (Figure 3A). The fluorescence intensity profiles and quantitative analysis of colocalization confirmed a significant increase in H4^PS1A431E^, H4^PS1E280A^, H4^PS1M146V^, and H4^PS1H163R^ cell lines after induction (Figure 3A-B). Additionally, we performed transmission electron microscopy (TEM) analysis in each H4^PS1^ cell line to further corroborate this result. Consistent with our colocalization data, the percentage of mitochondria with ER contacts was significantly increased in H4^PS1A431E^, H4^PS1E280A^, H4^PS1H163R^, and H4^PS1M146V^ cell lines after induction (Figure 3C-D).

### Mitochondrial functions are differentially altered in H4^PS1^ cell lines upon PS1 mutant induction

**Figure 4:**
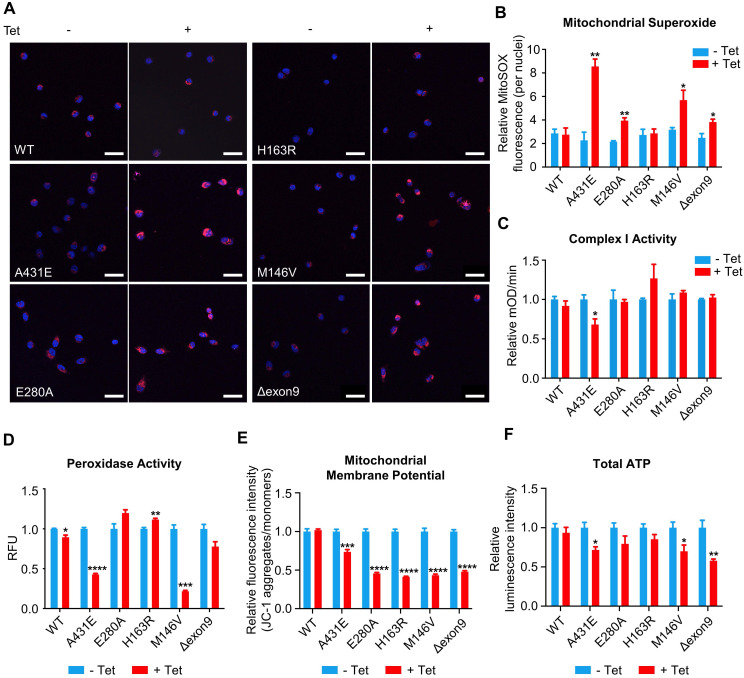
** Mitochondrial functions are differentially altered in H4^PS1^ cell lines upon induction. (A)** Representative confocal images of MitoSOX (red) and Hoechst (blue) stained H4^PS1^ cell lines before and after tetracycline (100 ng/ml) treatment. Scale bar, 50 µm. **(B)** Quantification of mitochondrial superoxide production in H4^PS1^ cell lines before and after tetracycline (100 ng/ml) treatment. For each independent imaging experiment, 25-51 cells were analyzed. n = 3; *P < 0.05*,* **P < 0.01; Student's *t*-test (two-tailed). **(C)** Quantification of mitochondrial complex I activity in H4^PS1^ cell lines before and after tetracycline (100 ng/ml) treatment using the complex I enzyme activity assay. n = 3; *P < 0.05; Student's *t*-test (two-tailed). **(D)** Quantification of peroxidase activity in H4^PS1^ cell lines before and after tetracycline (100 ng/ml) treatment using the peroxidase assay. n = 3; *P < 0.05*,* **P < 0.01, ***P < 0.001, ****P < 0.0001; Student's *t*-test (two-tailed). **(E)** Quantification of mitochondrial membrane potential in H4^PS1^ cell lines before and after tetracycline (100 ng/ml) treatment using the Mito-Probe JC-1 assay. n = 4; ***P < 0.001, ****P < 0.0001; Student's *t*-test (two-tailed). **(F)** Quantification of total ATP level in H4^PS1^ cell lines before and after tetracycline (100 ng/ml) treatment using the ATP bioluminescence detection assay. n = 4; *P < 0.05, **P < 0.01; Student's *t*-test (two-tailed). The values shown indicate the means ± SEM.

The majority of ROS are produced during mitochondrial respiration. This ROS production causes oxidative damage in numerous pathologies, including AD. In fact, oxidative damage has been observed in the early stage of AD, even before the onset of plaque and tau pathology 4. Thus, we first measured ROS production in each of H4^PS1^ cell lines before and after PS1 mutant induction using CM-H_2_DCFDA. All H4^PS1^ cell lines, except H4^PS1E280A^, exhibited an elevated ROS production after PS1 mutant induction (Figure S2). Since superoxide (O_2_^•-^) is the proximal mitochondrial ROS, we measured the production of O_2_^•-^ in each H4^PS1^ cell line. After induction, there was a significant increase in mitochondrial O_2_^•-^ in H4^PS1A431E^, H4^PS1E280A^, H4^PS1M146V^, and H4^PS1Δexon9^ cell lines based on MitoSOX fluorescence (Figure 4A-B). There are two sources of mitochondrial O_2_^•-^: Complex I (NADH Coenzyme Q oxidoreductase) and Complex III (ubiquinol cytochrome *c* oxidoreductase). Complex III is responsible for most O_2_^•-^ production in the heart and lung, while complex I is the main source of O_2_^•-^ in the brain 68-71. When complex I activity is impaired, NADH builds up and the reduction potential of NAD^+^ decreases, which leads to an enhanced superoxide production 72. In fact, this inverse relation between superoxide production and complex I activity has been previously reported 73-75. Thus, we examined whether the observed increase in mitochondrial O_2_^•-^ level was due to abnormal complex I activity. The H4^PS1A431E^ cell line exhibited significantly reduced complex I activity after induction (Figure 4C).

Superoxide dismutase (SOD) catalytically converts O_2_^•-^ generated during respiration to hydrogen peroxide (H_2_O_2_) and molecular oxygen (O_2_) 76. Accumulation of H_2_O_2_ can have a deleterious effect on cells because it can be converted to highly reactive hydroxyl radical (^•^OH) via the Fenton reaction in the presence of Fe^2+^ 76. Catalase, an antioxidant enzyme, prevents this from happening by converting H_2_O_2_ to H_2_O and O_2_. Unfortunately, catalase is not present in the mitochondria. In mitochondria, Glutathione Peroxidase (GPx), an enzyme with peroxidase activity, reduces H_2_O_2_ to H_2_O and lipid hydroperoxides to their corresponding alcohols to protect the organism from oxidative damage 76. Therefore, we examined whether peroxidase activity in each H4^PS1^ cell line was perturbed by PS1 mutant expression. After induction, H4^PS1A431E^ and H4^PS1M146V^ cell lines showed significantly impaired peroxidase activity (Figure 4D). Surprisingly, H4^PS1H163R^ cell line exhibited significantly increased peroxidase activity. Since complex I and peroxidase activities are essential for maintaining mitochondrial membrane potentials and ATP levels 77-79, we examined mitochondrial membrane potentials and ATP levels in H4^PS1^ cell lines before and after PS1 mutant induction. All five H4^PS1^ mutant cell lines exhibited significantly decreased mitochondrial membrane potential and ATP levels were significantly reduced in H4^PS1A431E^, H4^PS1M146V^, and H4^PS1Δexon9^ cell lines (Figure 4E-F). Previously, it has been reported that tetracycline treatment alone can disturb mitochondrial function 80. In order to confirm that the observed mitochondrial dysfunctions in each H4^PS1^ cell line were due to PS1 mutant induction, not tetracycline treatment, we assessed some of mitochondrial functions before and after tetracycline treatment in naïve H4 cells. 100 ng/ml of tetracycline treatment did not affect mitochondrial superoxide production, membrane potential, intracellular ROS production, and peroxidase activity (Figure S3A-D). Furthermore, the concentration of tetracycline used in the study by Moullan et al was five to ten times higher than the concentration used in the current study 80.

### PS1 mutant induction has little effect on mitochondrial bioenergetics

We next wondered whether *PS1* mutation affects mitochondrial respiration. We used a Seahorse XF24 extracellular flux analyzer to measure OCR during sequential treatment with compounds that modulate mitochondrial activity and examine the functional bioenergetic capacity of mitochondria in each H4^PS1^ cell line. There was no significant difference in basal OCR before and after the induction in most H4^PS1^ cell lines except for H4^PS1H163R^ cell line (Figure S4). After induction, H4^PS1H163R^ cell line exhibited significantly increased basal OCR (Figure S4). The basal respiration is composed of two components: the oxygen consumption devoted to ATP synthesis and oxygen consumption due to the natural proton leak across the inner mitochondrial membrane 81. The addition of oligomycin, an ATP synthase inhibitor, uncouples these two components. There was no significant difference in the ATP-linked respiration and proton leak in all H4^PS1^ cell lines before and after the induction (Figure S4).

### Gene expression profile in PS1M146V knock-in mouse is distinct from that of wild-type

**Figure 5:**
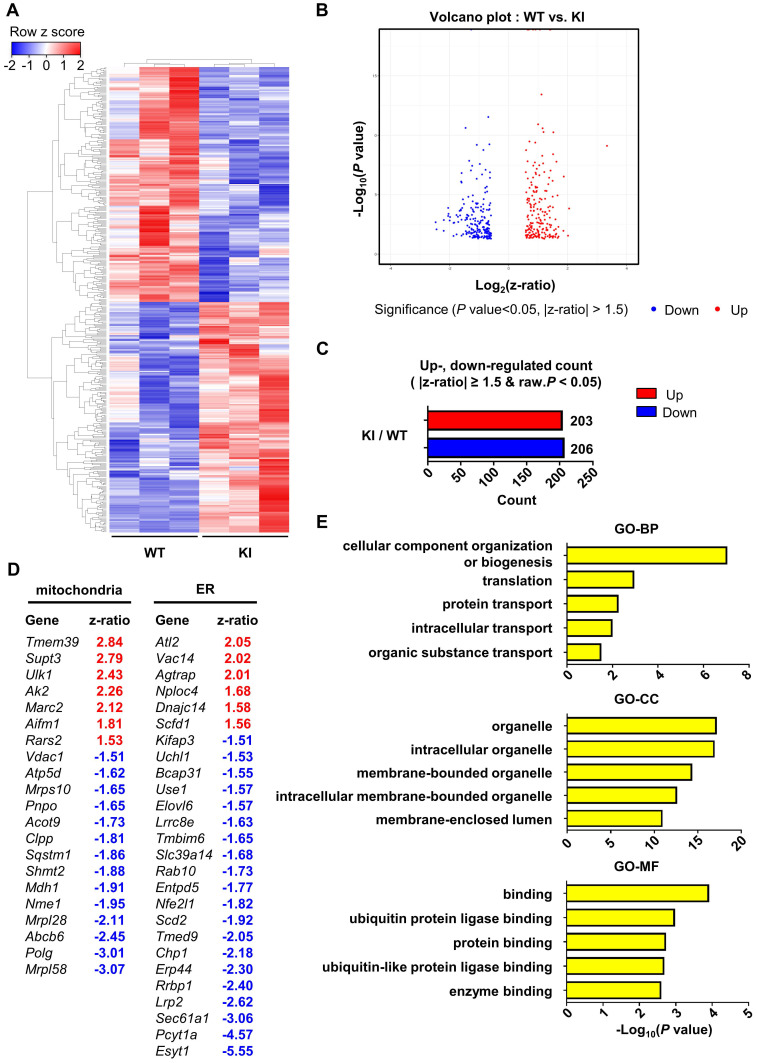
** Hippocampal gene expression profile of PS1M146V knock-in mouse is distinct from that of wild-type. (A)** Hierarchical cluster analysis and **(B)** volcano plot of differentially expressed genes (DEGs) in the hippocampal samples of PS1M146V knock-in and wild-type mice. **(C)** Numbers of differentially upregulated and down-regulated count by z-ratio and *P*-value. **(D)** Lists of DEGs related to either mitochondria or ER. **(E)** Gene Ontology and pathway enrichment analysis of DEGs. BP, biological processes; CC, cellular components; MF, molecular functions.

Finally, in order to examine the effect of PS1 mutant overexpression on gene expression profile, we performed microarray analysis of hippocampal samples from wild-type and PS1M146V knock-in mice. A total of 16,896 raw reads were obtained using mouse NIA 17k cDNA filters. To identify differentially expressed genes (DEGs), the raw reads were preprocessed and analyzed with DIANE 1.0. Eventually, a total of 409 DEGs were identified, of which 203 (49.63%) were upregulated, and 206 (50.37%) were down-regulated (Table S1). Hierarchical clustering showed that the gene expression profile of PS1M146V knock-in mouse is distinct from that of wild type (Figure 5A-C). We then identified 21 DEGs that are related to mitochondria and 26 DEGs that are associated with ER (Figure 5D). To gain further insight into the function of the DEGs, we used g:Profiler (ver 0.6.7) for performing the Gene Ontology (GO) analysis. The results of GO analysis revealed that the DEGs were significantly enriched in biological processes, such as 'cellular component organization or biogenesis', 'translation', 'protein transport', 'intracellular transport' and 'organic substance transport' (Figure 5E and Table S2). The DEGs were also significantly enriched in cellular components, such as 'organelle', 'intracellular organelle', 'membrane-bounded organelle', 'intracellular membrane-bounded organelle', and 'membrane-enclosed lumen' (Figure 5E and Table S2). Lastly, the DEGs were significantly enriched in molecular functions, such as 'binding', 'ubiquitin protein ligase binding', 'protein binding', 'ubiquitin-like protein ligase binding', and 'enzyme binding' (Figure 5E and Table S2). To confirm that the differential expression data was not driven by one particular animal of the three used for each genotype, we performed a principal component analysis (PCA). The separation between wild-type group and PS1M146V knock-in group was evident (Figure S5A). Furthermore, since presenilin 1 plays a significant role in the regulation of neuronal differentiation via affecting notch signaling pathway, we examined whether there were changes in cell-type proportions in the brains of PS1M146V knock-in mice 82. We analyzed expression levels of genes encoding specific markers for neurons, microglia, and astrocytes. All markers had z-ratios less than 1.3 and greater than -1.3 with *P* values greater than 0.10 indicating proportions of neurons, microglia, and astrocytes in the brain did not change in PS1M146V knock-in mice (Figures S5B).

### ATL2 is involved in abnormally elevated ER-mitochondria contacts in H4^PS1M146V^ cell line after PS1 mutant induction

**Figure 6:**
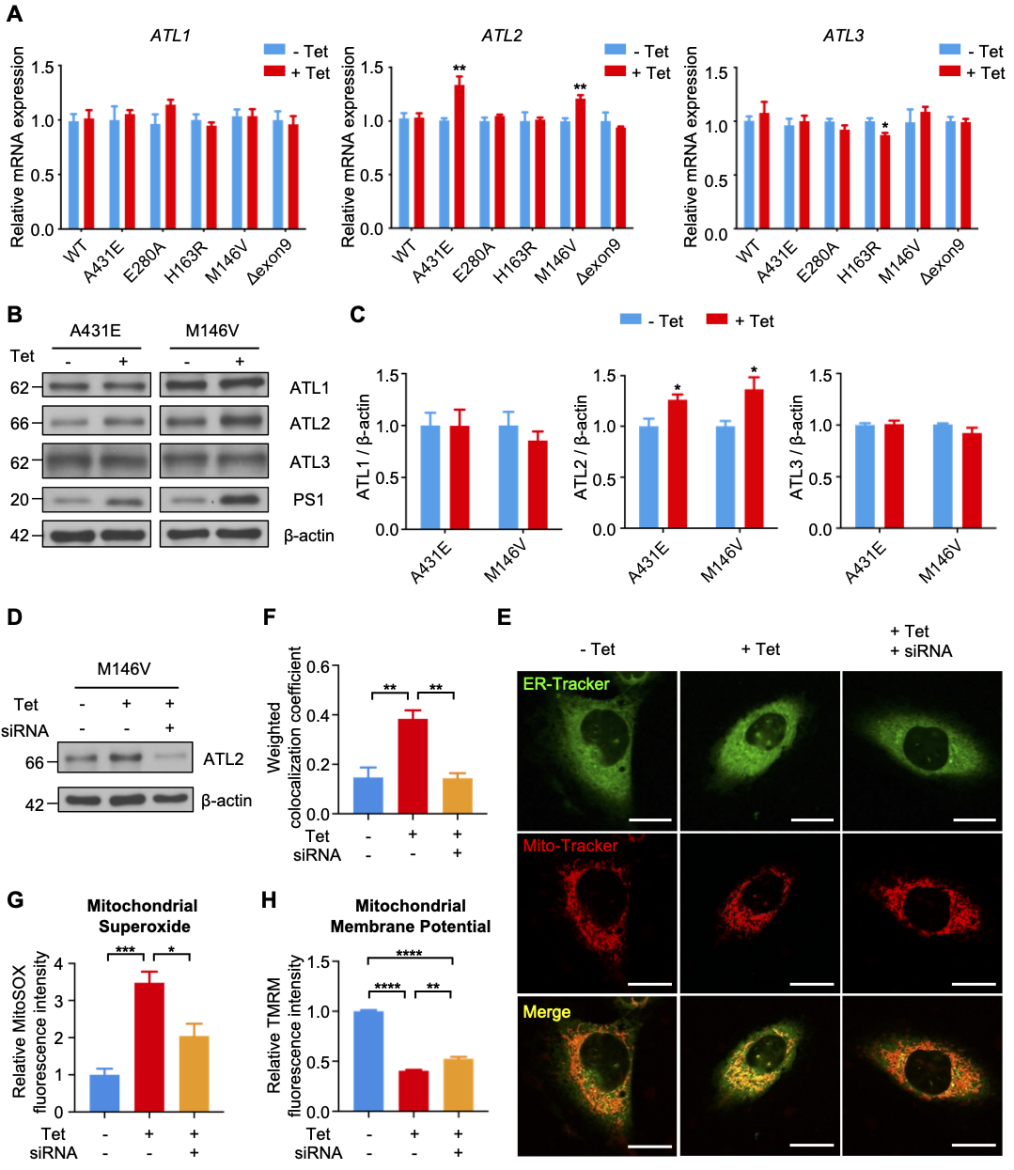
** Knockdown of ATL2 after PS1 mutant induction rescues abnormally elevated ER-mitochondria colocalization back to normal level in the H4^PS1M146V^ cell line. (A)** mRNA expression levels of *ATL1*,* ATL2*, and *ATL3* in H4^PS1^ cell lines before and after tetracycline (100 ng/ml) treatment. **(B)** Representative western blots of ATL1, ATL2, and ATL3 in H4^PS1A431E^ and H4^PS1M146V^ cell lines before and after tetracycline (100 ng/ml) treatment. **(C)** Quantification of ATL1, ATL2, and ATL3 expressions in (B). n = 3; *P < 0.05; Student's *t*-test (two-tailed). **(D)** A representative western blot of ATL2 in H4^PS1M146V^ cell line before and after tetracycline (100 ng/ml) treatment or siRNA (40 nM) transfection. **(E)** Representative confocal images of Mito-Tracker (red) and ER-Tracker (green) labeled H4^PS1M146V^ cell line before and after tetracycline (100 ng/ml) treatment or siRNA (40 nM) transfection. Scale bar, 20 µm. **(F)** The weighted colocalization coefficient between mitochondria and ER. For each independent imaging experiment, 10-15 cells were analyzed. n = 3; **P < 0.01; One-way ANOVA with Tukey post-hoc test. **(G)** Quantification of mitochondrial superoxide production in H4^PS1M146V^ cell line before and after tetracycline (100 ng/ml) treatment or siRNA (40 nM) transfection. n = 4; *P < 0.05*,* ***P < 0.001; One-way ANOVA with Tukey post-hoc test. **(H)** Quantification of mitochondrial membrane potential in H4^PS1M146V^ cell line before and after tetracycline (100 ng/ml) treatment or siRNA (40 nM) transfection using the TMRM probe. n = 4; **P < 0.01, ****P < 0.0001. The values shown indicate the means ± SEM.

Among 26 DEGS related to ER, we focused on *Atl2*, which was the most upregulated ER-related genes in PS1M146V knock-in mice. *Atl2* encodes an ER-resident membrane-bound GTPase Atlastin 2 (ATL2), which mediates ER membrane fusion and tethering. We first examined whether PS1 mutant induction increases ATL2 expression in each H4^PS1^ cell line. While there was no significant difference in *ATL1* and *ATL3* mRNA expression, *ATL2* mRNA expression was significantly increased in H4^PS1A431E^ and H4^PS1M146V^ cell lines after induction (Figure 6A). Consistent with qPCR data, ATL2 protein expression was also significantly increased in H4^PS1A431E^ and H4^PS1M146V^ cell lines after PS1 mutant induction while ATL1 and ATL3 protein expression did not change (Figure 6B-C). Since ATL2 is involved in ER membrane fusion and tethering, which is critical in forming ER-mitochondrial contacts, we hypothesized that ATL2 might play a crucial role in ER-mitochondria interaction by physically increasing ER-mitochondria tethering. In fact, a previous study has identified ATL2 as a protein associated with ER-mitochondria contacts 83. In order to test our hypothesis, we examined whether knockdown of ATL2 after PS1 mutant induction rescues elevated ER-mitochondria colocalization back to normal level in H4^PS1M146V^ cell line. We first confirmed siRNA-mediated ATL2 knockdown via western blot (Figure 6D). We then utilized confocal microscopy to observe ER-mitochondria colocalization. PS1M146V induction significantly increased ER-mitochondria colocalization while knocking down ATL2 rescued an elevated ER-mitochondria colocalization back to normal level (Figure 6E-F). Furthermore, we investigated whether knocking down ATL2 can rescue some of mitochondrial dysfunctions observed in H4^PS1M146V^ cell line upon induction. In fact, the abnormally elevated mitochondrial superoxide level in induced H4^PS1M146V^ cell line was rescued when ATL2 was knocked down (Figure 6G). Although, compromised mitochondrial membrane potential in induced H4^PS1M146V^ cell line was not rescued back to normal level, knocking down ATL2 could significantly increase mitochondrial membrane potential (Figure 6H).

### ATL2 expression level is elevated in the brains of both the AD mouse model and AD patients

**Figure 7:**
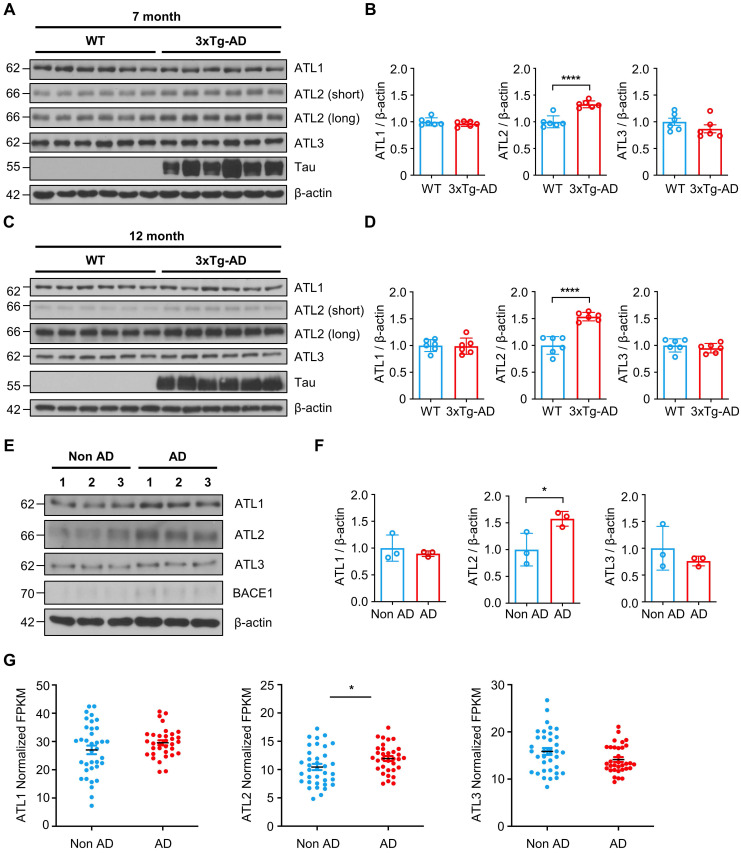
** ATL2 expression level is elevated in the brains of AD mouse model and AD patients. (A)** Representative western blots of ATL1, ATL2, ATL3, and Tau in the hippocampi of seven-month-old 3xTg-AD mice (male, n = 2; female, n = 4) and age-matched wild-type (male, n = 2; female, n = 4). **(B)** Quantification of ATL1, ATL2, and ATL3 expressions in (A); ****P < 0.0001; Student's *t*-test (two-tailed). **(C)** Representative western blots of ATL1, ATL2, ATL3, and Tau in the hippocampi of twelve-month-old 3xTg-AD mice (male, n = 2; female, n = 4) and age-matched wild-type (male, n = 2; female, n = 4). **(D)** Quantification of ATL1, ATL2, and ATL3 expressions in (C); ****P < 0.0001; Student's *t*-test (two-tailed). **(E)** Representative western blots of ATL1, ATL2, ATL3, and BACE1 in the inferior parietal lobule of AD patients (n = 3) and age-matched control subjects (n = 3). **(F)** Quantification of ATL1, ATL2, and ATL3 expressions in (C); *P < 0.05; Student's *t*-test (two-tailed). **(G)** Expression levels of *ATL1*,* ATL2*, and *ATL3* in the frontal white matter of subjects with (red, n = 33) and without (blue, n = 36) AD; *P < 0.05; Student's *t*-test (two-tailed). The values shown indicate the means ± SEM.

Lastly, we evaluated ATL2 expression levels in the brains of both the AD mouse model and AD patients. Since ATL2 expression level was elevated in hippocampi of PS1M146V knock-in mice as well as in H4^PS1M146V^ cell line upon induction, we examined ATL2 expression level in the brains of 3xTg-AD mice, which harbor PS1M146V mutation. ATL2 was significantly upregulated in hippocampi of both seven-month and twelve-month-old 3xTg-AD mice compared to those of age-matched wild-type mice, while there was no significant difference in the expression of ATL1 and ATL3 (Figure 7A-D). Next, we examined whether ATL2 expression level is also elevated in AD patients. The ATL2 expression level was measured in rapidly autopsied specimens from the inferior parietal lobule of AD patients and age-matched control subjects. The age, sex, time post-mortem interval, and amyloid plaque counts can be found in Table S3. The ATL2 expression level was significantly elevated in AD patients, while there was no significant difference in the expression level of ATL1 and ATL3 (Figure 7E-F). Additionally, in order to confirm the gene expression level of *ATL2* in the brains of AD patients, we utilized publicly available data from the Allen Institute for Brain Science (Aging, Dementia, and TBI Study). The study contained clinical data from 107 subjects, of which 57 were free from dementia, and 50 were clinically diagnosed with dementia. Among 57 non-dementia subjects, we excluded any individuals with one of the following conditions: (1) Braak stage 6, (2) NIA-Reagan stage 3, (3) three or more traumatic brain injuries (TBIs). This resulted in 36 subjects (Non-AD). Among 50 subjects that were clinically diagnosed with dementia, 43 met NINDS-ARDA Alzheimer's criteria for either 'Probable AD' or 'Possible AD'. Of these 43 AD subjects, we excluded any individuals with one of the following conditions: (1) Braak stage 0, (2) NIA-Reagan stage 0, (3) three or more TBIs. This resulted in 33 subjects (AD). To determine whether *ATL2* gene expression is elevated in AD subjects, we analyzed RNA-seq datasets. Within frontal white matter (FWM), significantly increased expression levels were observed in AD subjects for *ATL2* but not for *ATL1* or *ATL3* (Figure 7G).

## Discussion

We used both biochemical and imaging approaches to demonstrate the effect of *PS1* mutations on mitochondrial functions in six different H4^PS1^ cell lines. It is well established that PS1 localizes to various subcellular compartments, including ER, MAMs, and mitochondria 67. Here, we focused on the effect of five FAD-linked *PS1* mutations on mitochondrial functions.

**Figure 8:**
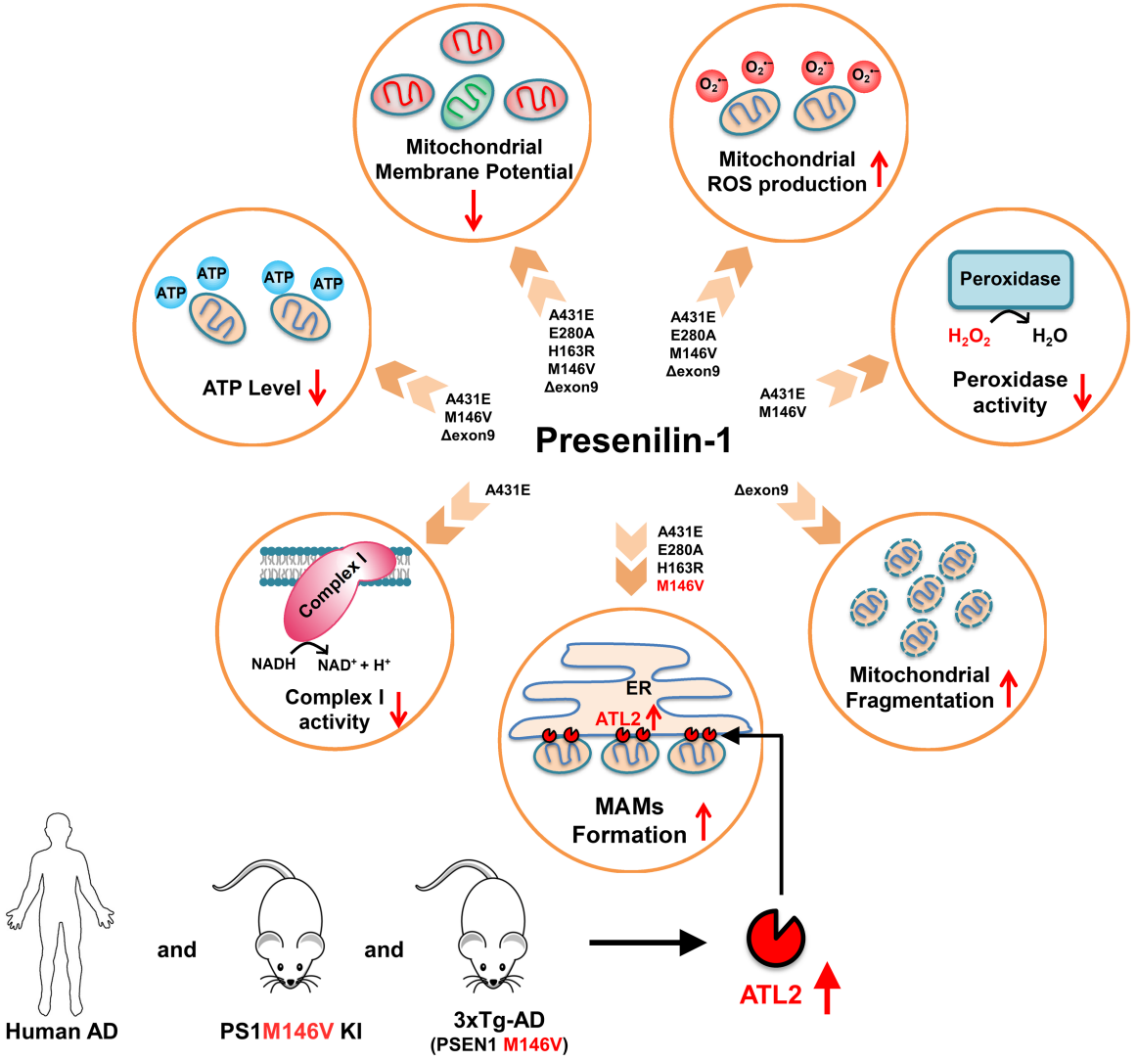
** A schematic diagram of PS1 mutant type-dependent mitochondrial dysfunctions.** Each PS1 mutant results in a different degree of mitochondrial dysfunctions. Most importantly, A431E, E280A, H163R, and M146V mutants induced an increase in ER-mitochondria interactions. The comparative profiling of hippocampal gene expression in PS1M146V knock-in mice revealed that PS1M146V upregulates ATL2 expression level, which increases ER-mitochondria contacts. Moreover, ATL2 expression levels were significantly elevated in the brains of 3xTg-AD mice and AD patients.

First, we report an increased ER-mitochondria contact in H4^PS1A431E^, H4^PS1E280A^, H4^PS1H163R^, and H4^PS1M146V^ cell lines after induction (Figure 8 and Table S4). Consistent with our results, studies have shown increased ER-mitochondria connectivity in human fibroblasts isolated from individuals with FAD mutations and individuals with SAD 67. Moreover, post-mortem analysis of human AD brains and AD mouse models' brains also revealed an increase in ER-mitochondria connectivity 84. Furthermore, this observation has also been reported in a transgenic AD mouse model expressing mutant tau P301L and in both human and mouse fibroblasts exposed to apolipoprotein E (ApoE4), a major risk factor for developing SAD 85, 86. However, a precise mechanism behind how PS1 regulates MAM formation has not been revealed yet. Recently, Pera et al reported that unprocessed APP-CTFs, specifically C99, are accumulated in MAM regions, resulting in an elevated sphingolipid turnover and an altered lipid composition of both MAM and mitochondrial membranes 87. Thus, they suggested that this increased localization of C99 in MAMs in AD might be responsible for the upregulation of ER-mitochondrial connections. However, a previous study has shown that catalytic activity of γ-secretase has little effect on establishing ER-mitochondrial connectivity 67. Thus, a precise mechanism behind elevated ER-mitochondrial connections in AD still remains elusive. Although it is unclear how PS1 regulates MAM formation, our findings and previous reports may suggest abnormal MAMs formation as a common denominator underlying both SAD and FAD pathogenesis.

We also report increased mitochondrial ROS production in H4^PS1A431E^, H4^PS1E280A^, H4^PS1M146V^, and H4^PS1Δexon9^ cell lines after induction (Figure 8 and Table S4). This increase in mitochondrial ROS production has been reported in both DT40 and PC12 cells expressing PS1M146L mutation 88. The observed increase in mitochondrial ROS production is partly due to the impaired mitochondrial electron transport chain and dysfunctional antioxidant enzyme. Complex I activity was significantly decreased in the H4^PS1A431E^ cell line, and the activity of peroxidase, an antioxidant enzyme in mitochondria, was compromised in H4^PS1A431E^ and H4^PS1M146V^ cell lines after induction (Figure 8 and Table S4). A previous study has reported that mutations in *C. elegans* gene encoding a PS1 homolog, *sel-12*, result in elevated ER-mitochondria contacts leading to increased mitochondrial calcium uptake and ROS production 89. We have also observed increased ER-mitochondria contacts and mitochondrial ROS in H4^PS1A431E^, H4^PS1E280A^, and H4^PS1M146V^ cell lines after induction. The influx of calcium to the mitochondrial matrix affects multiple aspects of mitochondrial functions such as ATP synthesis, mitochondrial permeability transition pore opening, and the mitochondrial membrane potential which ultimately leads to mitochondrial ROS production. In fact, total ATP level was significantly reduced in H4^PS1A431E^, H4^PS1M146V^, and H4^PS1Δexon9^ cell lines and the mitochondrial membrane potential was abrogated in all H4^PS1^ cell lines (Figure 8 and Table S4). Thus, calcium could be a potential mediator of mitochondrial ROS production in H4^PS1A431E^, H4^PS1E280A^, and H4^PS1M146V^ cell lines. It is not clear why some of the PS1 mutants caused oxidative stress, whereas others did not. This could be explained by the fact that neurons generate much higher amounts of ROS compared to the cell lines examined in the present study. It will, therefore, be important to determine if and how each *PS1* mutation affects oxidative stress and mitochondrial function in neurons.

Surprisingly, despite the increased ER-mitochondrial contacts or mitochondrial ROS production, the size and the shape of mitochondria in H4^PS1A431E^, H4^PS1E280A^, H4^PS1H163R^, and H4^PS1M146V^ cell lines did not change after induction. On the other hand, mitochondria in the H4^PS1Δexon9^ cell line were shortened and spherical, which indicates mitochondrial fragmentation (Figure 8 and Table S4). However, the expression level of DRP1 was significantly decreased in H4^PS1Δexon9^ cell line. The reduced levels of DRP1 have been previously reported in brain samples from AD patients as well as in fibroblasts from both sporadic and familial AD patient 58,90. Furthermore, predominant mitochondrial fragmentation and decreased levels of DRP1 upon overexpression of APP in M17 neuroblastoma cells have been reported by the same group 91. Moreover, decreased levels of DRP1 have also been observed in astrocytes expressing ApoE4, a significant risk factor for sporadic AD 92. Additionally, mitochondria-localized DRP1 has been reported to be reduced in sporadic AD 93. Along with DRP1, FIS1 is another protein that promotes mitochondrial fission. In fact, it has been reported that FIS1 can mediate mitochondrial fragmentation in the absence of DRP1 by binding to MFN2 and OPA1 and inhibiting their GTPase activity, which blocks the mitochondrial fusion machinery 94. Thus, FIS1 could be responsible for the increased mitochondrial fragmentation in H4^PS1Δexon9^ cell line upon induction.

Lastly, we report a distinct hippocampal gene expression profile of PS1 mutant (M146V) knock-in mouse compared to that of wild-type mouse. We identified a total of 409 DEGs of which 21 were related to mitochondria and 26 were related to ER. Since PS1 is mostly localized to ER and Golgi, the DEGs were significantly enriched in biological processes, such as 'translation', 'protein transport', 'intracellular transport', and 'organic substance transport'. Additionally, the DEGs were also significantly enriched in cellular components, such as 'organelle', 'intracellular organelle', 'membrane-bounded organelle', and 'membrane-enclosed lumen'. This suggests that PS1 might have a significant role in the endomembrane system.

Among ER-related DEGs, *Atl2*, a gene that encodes ER-shaping protein ATL2, was the most upregulated gene. ATL2 regulates ER structure and plays a critical role in ER membrane fusion and tethering, which are crucial for the formation of ER-mitochondria contact sites. In fact, down-regulation of ATL2 after PS1 mutant induction rescued abnormally elevated ER-mitochondria colocalization back to a normal level suggesting a crucial role of ATL2 in the formation of ER-mitochondria contact sites. Moreover, significantly elevated ATL2 expression levels were observed in the brains of both 3xTg-AD mice and AD patients.

Although it is unclear how PS1 mutant regulates ATL2 expression level, PS1 might play an important role in maintaining and regulating MAMs structure formation through ATL2, which is significantly related to AD pathogenesis. Therefore, discovering the precise role of PS1 and ATL2 in MAMs formation might open a new therapeutic window for AD treatment.

## Conclusions

Overall, our findings suggest that each of five *PS1* mutations have detrimental effect on different mitochondrial functions. Most importantly, most of *PS1* mutations increased ER-mitochondria contacts and mitochondrial ROS production while reducing mitochondrial membrane potential. We also suggest that PS1 might play a critical role in maintaining and regulating ER-mitochondria interactions through ATL2, an ER-shaping protein whose expression level is significantly elevated in brains of both FAD mouse model and AD patients. We believe that our discovery of the effects of *PS1* mutations on mitochondria function and ER-mitochondria interactions will have significant clinical and biological implications in pathology of AD. However, further studies are needed to elucidate how PS1 mutant regulates ATL2 expression level.

## Supplementary Material

Supplementary figures and tables.

Supplementary table S1.

Supplementary table S2.

## Abbreviations

Aβ: β-amyloid
ACT: Adult Change in Thought
AD: Alzheimer's disease
ApoE4: apolipoprotein E
APP: amyloid precursor protein
APP-CTF: amyloid precursor protein C-terminal fragment
ATL2: Atlastin-2
EOFAD: early-onset familial Alzheimer's disease
DCF: 2′,7′-dichlorofluorescein
DEGs: differentially expressed genes
DRP1: dynamin-related protein 1
ER: endoplasmic recticulum
FAD: familial Alzheimer's disease
FWM: frontal white matter
GO: Gene Ontology
GPx: Glutathione Peroxidase
H2DCF-DA: 2′,7′-dichlorofluorescein diacetate
H_2_O_2_: hydrogen peroxide
MAMs: mitochondria-associated-membranes
MFN2: mitofusin 2
mtDNA: mitochondrial DNA
O_2_: molecular oxygen
O_2_^•-^: superoxide
OCR: oxygen consumption rate
OPA1: optic atrophy1
^•^OH: hydroxyl radical
PS1: presenilin-1
ROS: reactive oxygen species
SAD: sporadic Alzheimer's disease
SOD: superoxide dismutase
TBIs: traumatic brain injuries
TEM: transmission electron microscopy
