# Supplementary material for: Alzheimer's disease-causing presenilin-1 mutations have deleterious effects on mitochondrial function
Source: Theranostics. 2021 Aug 17;11(18):8855–73. doi: 10.7150/thno.59776 (PMC8419044; doi:10.7150/thno.59776)
Supplement: Supplementary file 1 — Supplementary figures and tables. [file thnov11p8855s1.pdf]

Alzheimer's disease-causing presenilin-1 mutations have deleterious effects on mitochondrial function

Supplementary materials

Table S1. A list of all DEGs. (XLSX)

Table S2. A list of significantly enriched GO terms. (XLSX)

Table S3. Clinical data on AD patients and non-AD control subjects.

Table S4. Summary of mitochondrial dysfunction observed in $H4^{PS1}$ cell lines.

Figure S1. $H4^{PS1\Delta exon9}$ cell line exhibits mitochondria aggregation upon induction.

Figure S2. Intracellular ROS production is elevated in $H4^{PS1}$ cell lines.

Figure S3. Tetracycline does not affect mitochondrial function in $H4^{PS1}$ cell lines.

Figure S4. PS1 mutant induction has little effect on mitochondrial bioenergetics.

Figure S5. Cell-type proportions in the brains of PS1M146V knock-in mice are not altered.

Supplementary Tables and Figures

Table S3. Clinical data on AD patients and non-AD control subjects. The number of neuritic plaques per 2.35 mm^2 microscopic field were counted in Aβ antibody-stained sections of inferior parietal lobule. The value for each subject is the mean of 5 most involved fields in each section. PMI = post-mortem interval.

Patients	Age (years)	Gender	PMI (h)	Neuritic plaques	Cause of Death	Braak stage
**Control**						
1	86	Female	2.25	7.6	Unknown	2
2	91	Female	4.00	10.4	Unknown	1
3	86	Female	3.75	7.8	Cardiovascular disease	1
Mean ± S.D.	87.7 ± 2.9		3.33 ± 0.95	8.6 ± 1.6		
**Alzheimer's disease**						
1	86	Female	4.25	23.4	Bowel obstruction	6
2	75	Female	2.33	19.0	Congestive heart failure	6
3	84	Male	4.50	34.8	Unknown	6
Mean ± S.D	81.7 ± 5.9		3.69 ± 1.19	25.7 ± 8.2		

Table S4. Summary of mitochondrial dysfunction observed in H4^{PS1} cell lines.

Mutation	Fragmentation	Formation of MAM	$O_2^{\bullet-}$ production	Complex I activity	Peroxidase activity	Mitochondrial membrane potential	ATP Level
A413E	-	↑	↑	↓	↓	↓	↓
E280A	-	↑	↑	-	-	↓	-
H163R	-	↑	-	-	↑	↓	-
M146V	-	↑	↑	-	↓	↓	↓
Δexon9	↑	-	↑	-	-	↓	↓

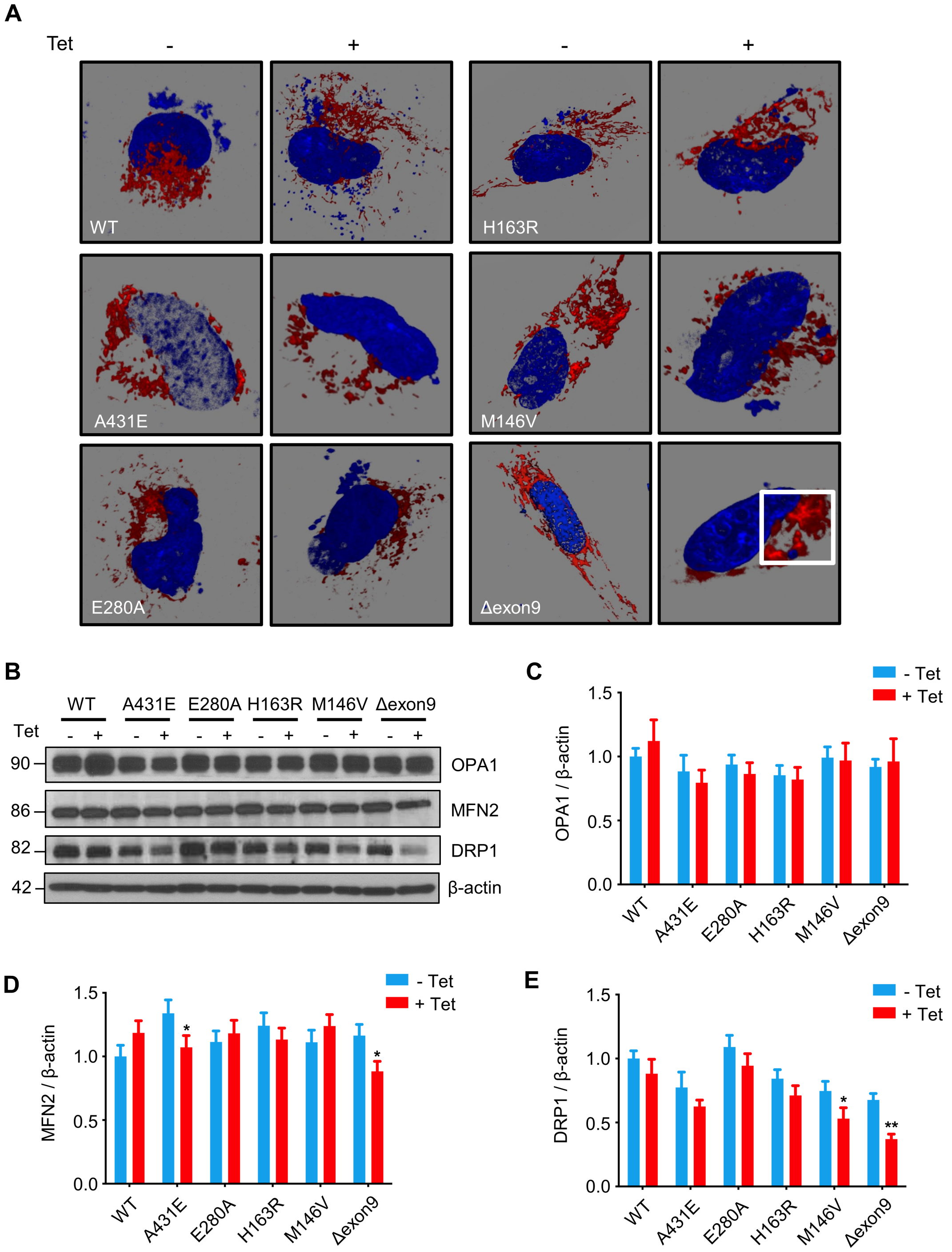

Figure S1. H4^{PS1Δexon9} cell line exhibits mitochondria aggregation upon induction. (A) Mitochondrial distribution observed using confocal microscopy z-stack imaging (z-axis:5μm, 100 slices). **(B)** Western blot of mitochondrial dynamics related proteins in H4^{PS1} cell lines. **(C-E)** Quantification of OPA1, MFN2, DRP1 expression levels in H4^{PS1} cell lines before and after tetracycline (100 ng/ml) treatment. n =3; *$P < 0.05$, **$P < 0.01$; Student's *t*-test (two-tailed). The values shown indicate the means ± SEM.

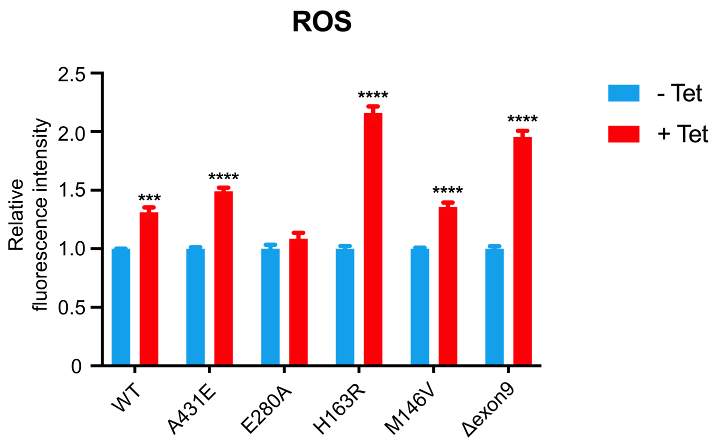

Figure S2. Intracellular ROS production is elevated in H4^{PS1} cell lines. Quantification of ROS production measured in H4^{PS1} cell lines before and after tetracycline (100 ng/ml) treatment. n = 4; ***$P < 0.001$, ****$P < 0.0001$; Student's *t*-test (two-tailed). The values shown indicate the means ± SEM.

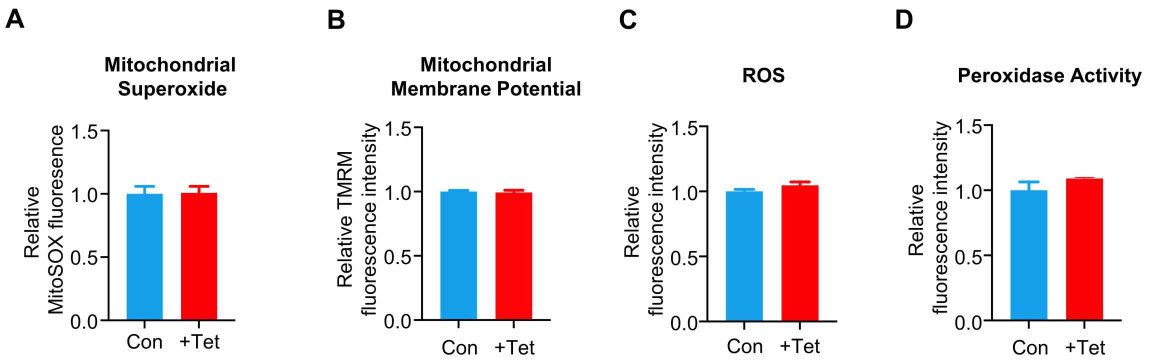

Figure S3. Tetracycline does not affect mitochondrial function in H4^{PS1} cell lines. (A) Quantification of mitochondrial superoxide production in H4^{PS1} cell lines before and after tetracycline (100 ng/ml) treatment using MitoSOX. n = 3; Student's *t*-test (two-tailed). **(B)** Quantification of mitochondrial membrane potential in H4^{PS1} cell lines before and after tetracycline (100 ng/ml) treatment using TMRM. n = 4; Student's *t*-test (two-tailed). **(C)** Quantification of ROS production in H4^{PS1} cell lines before and after tetracycline (100 ng/ml) treatment using CM-H_2DCFDA. n = 4; Student's *t*-test (two-tailed). **(D)** Quantification of peroxidase activity in H4^{PS1} cell lines before and after tetracycline (100 ng/ml) treatment using the peroxidase assay. n = 4; Student's *t*-test (two-tailed). The values shown indicate the means ± SEM.

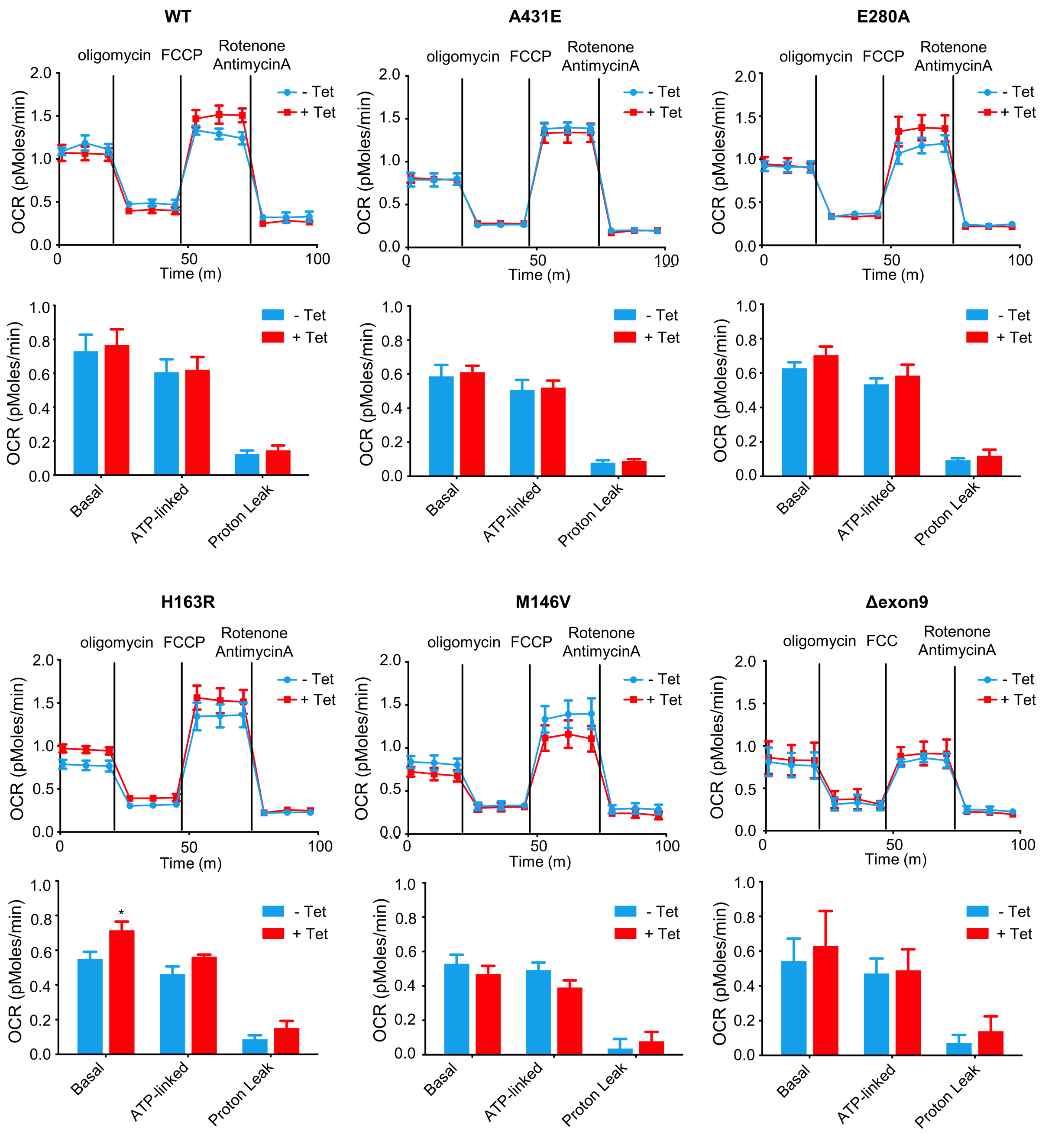

Figure S4. PS1 mutant induction has little effect on mitochondrial bioenergetics. Representative Seahorse oxygen consumption assay in $H4^{PS1}$ cell lines before and after tetracycline (100 ng/ml) treatment. Basal respiration, ATP-linked respiration, and proton leak measured in $H4^{PS1}$ cell lines before and after tetracycline (100 ng/ml) treatment. n = 4; *P < 0.05; Student's t-test (two-tailed). The values shown indicate the means ± SEM.

A

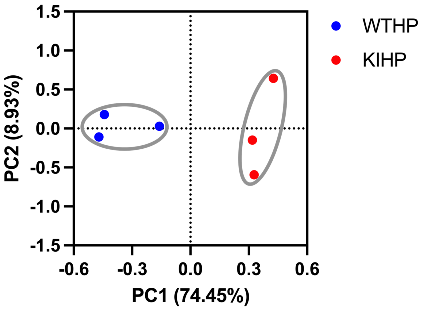

B

Neuron

Gene	z-ratio	*P* value
Calb1	0.11	0.33
Tubb3	-0.55	0.33
Mapt	0.17	0.90
Nefl	0.18	0.59

Microglia

Gene	z-ratio	*P* value
HexB	-1.29	0.12
Lgals3	-0.40	0.64
Csf1r	0.13	0.64

Astrocyte

Gene	z-ratio	*P* value
Slc1a3	0.59	0.26
Gja1	0.19	0.71
Aldo3	0.35	0.62

Figure S5. Cell-type proportions in the brains of PS1M146V knock-in mice are not altered. (A) A principal component analysis score plots from hippocampi of WT and PS1M146V knock-in mice. **(B)** Analysis of genes encoding specific markers for neurons, microglia, and astrocytes.
